# Combining arterial blood contrast with BOLD increases fMRI intracortical contrast

**DOI:** 10.1002/hbm.26227

**Published:** 2023-02-10

**Authors:** Nikos Priovoulos, Icaro Agenor Ferreira de Oliveira, Benedikt A. Poser, David G. Norris, Wietske van der Zwaag

**Affiliations:** ^1^ Spinoza Center for Neuroimaging Royal Netherlands Academy of Arts and Sciences Amsterdam The Netherlands; ^2^ Computational Cognitive Neuroscience and Neuroimaging Netherlands Institute for Neuroscience Amsterdam The Netherlands; ^3^ Experimental and Applied Psychology VU University Amsterdam The Netherlands; ^4^ MR‐Methods Group, Maastricht Brain Imaging Center, Faculty of Psychology and Neuroscience Maastricht University Maastricht the Netherlands; ^5^ Donders Institute for Brain, Cognition and Behaviour Radboud University Nijmegen Nijmegen The Netherlands; ^6^ Erwin L. Hahn Institute for MRI University of Duisburg‐Essen Essen Germany

**Keywords:** 3D‐EPI, arterial blood contrast, cerebral blood volume, layer‐dependent fMRI, magnetization prepared fMRI, ultrahigh field MRI

## Abstract

BOLD fMRI is widely applied in human neuroscience but is limited in its spatial specificity due to a cortical‐depth‐dependent venous bias. This reduces its localization specificity with respect to neuronal responses, a disadvantage for neuroscientific research. Here, we modified a submillimeter BOLD protocol to selectively reduce venous and tissue signal and increase cerebral blood volume weighting through a pulsed saturation scheme (dubbed Arterial Blood Contrast) at 7 T. Adding Arterial Blood Contrast on top of the existing BOLD contrast modulated the intracortical contrast. Isolating the Arterial Blood Contrast showed a response free of pial‐surface bias. The results suggest that Arterial Blood Contrast can modulate the typical fMRI spatial specificity, with important applications in in‐vivo neuroscience.

## Graphical abstract

BOLD fMRI is widely used in neuroscience, but has limited spatial specificity while alternative approaches based on tissue cerebral blood volume (CBV) change have limited sensitivity. Here, we combine temporally efficient saturation with center‐slice‐out readouts and BOLD‐correction to isolate arterial CBV for high‐resolution 7 T human fMRI. Our novel sequence shows improved specificity compared to BOLD, suggesting Arterial Blood Contrast to be a promising fMRI approach.

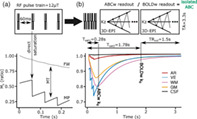

## 1 INTRODUCTION

fMRI has transformed human neuroscience by allowing the noninvasive visualization of deoxyhemoglobin concentration changes in relation to human behavior (Glover, 2011). Current state‐of‐the‐art applications have employed the increased signal and contrast‐to‐noise of ultrahigh field (UHF) MRI to reach submillimeter resolution, promising the examination of cortical layers and columns (Feinberg et al., 2018; Guidi et al., 2016; Polimeni et al., 2010; Vizioli & Muckli, 2015; Yacoub et al., 2008) and the distinction of cortical input/output and the signal directionality between brain regions (de Hollander et al., 2021; Sharoh et al., 2019). This important target is hindered by an implicit spatial specificity bias in the typical BOLD fMRI implementation and the limited sensitivity of other approaches, such as Vascular Space Occupancy (VASO), Arterial Spin Labelling (ASL), or spin‐echo‐based approaches (de Zwart et al., 2021; Kay et al., 2019; Lu & van Zijl, 2012; Norris, 2012). Here, we implement a pulsed saturation approach to increase intracortical fMRI contrast in humans while retaining high sensitivity and spatial resolution.

Typically, BOLD fMRI relies on a *T*_2_*w, gradient‐echo‐based EPI readout, due to its high signal‐to‐noise and efficient sampling rate. Changes in cerebral blood volume (CBV), flow (CBF), and oxygen metabolism during the performance of the task reduce the deoxyhemoglobin concentration that acts as an endogenous paramagnetic agent (Ogawa et al., 1990). The BOLD sensitivity is maximized at echo times close to the intravenous and tissue *T*_2_*. The venous signal then tends to dominate the BOLD contrast, resulting in a cortical‐depth dependent profile, since the venous anatomy of the isocortex is characterized by ascending veins pooling blood towards the larger pial veins (Duvernoy et al., 1981; Menon et al., 1995; Uludag et al., 2009). This introduces a spatial bias of the BOLD signal towards the pial surface and downstream of the neuronal activation (Boxerman et al., 1995; Norris, 2006).

Several approaches with increased spatial specificity have been suggested that combine the fast EPI sampling with magnetization preparation modules that weigh the signal towards CBV (Huber et al., 2018; Lu et al., 2003), CBF (Detre et al., 1994; Ivanov et al., 2017; Kashyap et al., 2021) or *T*_2_ changes around small vessels (*T*_2_‐prep) rather than intravascular changes (Lee et al., 2021; Parrish & Hu, 1994; Pfaffenrot et al., 2021; Saeed et al., 2014; Stunden et al., 2001). For such techniques, the implicit *T*_2_*‐weighting of the EPI is a contaminant to be reduced either with a normalization step or with center‐out readout schemes that minimize echo time (TE) and thus decrease *T*_2_*‐contrast buildup (Detre et al., 1994; Kurban et al., 2022; Lu et al., 2003; Pfaffenrot et al., 2021). Such approaches can increase the fMRI spatial specificity and have therefore found applications in high‐resolution layer fMRI studies (Yu et al., 2021), albeit with an implicit tradeoff between sensitivity, specificity, and the availability of the method. Similarly, spin‐echo (Goense & Logothetis, 2006; Yacoub et al., 2005) and gradient‐echo‐and‐spin‐echo approaches (Feinberg et al., 1995; Moerel et al., 2018; Oshio & Feinberg, 1991; Scheffler et al., 2021) have been successfully demonstrated, though again suffering from decreased sensitivity compared to BOLD. In practice, in the cognitive or clinical neuroscientific domains where there is a demand for simultaneous high spatial and temporal resolution and coverage, non‐BOLD approaches have seen limited applications (though this may change in the future, e.g., Finn et al., 2019; Oliveira et al., 2022).

Arterial‐specific CBV‐weighted approaches have been suggested in the past as a potentially high‐specificity approach, due to the sizeable CBV change in the arterioles (Kennerley et al., 2010) but not in surface arteries (Uludag & Blinder, 2018). Such approaches rely on either ASL variants (potentially requiring the individual optimization of tagging duration; Jahanian et al., 2015) or the preferential signal reduction of the tissue and venous compartments over the arteries (Kim et al., 2008; Kim & Kim, 2010; Vazquez et al., 2006). This signal reduction is achieved through the attenuation of the signal of the water protons bound to the macromolecules in the brain, such as the myelin lipids found in white (WM) and gray matter (GM), and the transfer of spin‐coherence to the free‐water protons of the tissue (magnetization transfer; MT; Henkelman et al., 1993). The venous compartment can be further selectively saturated with minimal effects on arterial and free‐water signal due to its distinct *T*_2_ value (Cai et al., 2013). Such approaches can provide an arterial‐based signal (Kim et al., 2008) with increased intracortical specificity due to the arteriole and capillary dilation, as shown in cats (Kim & Kim, 2010). Recently, an efficient saturation‐based arterial CBV‐weighted method was introduced at 3 T, relying on a short echo time to reduce BOLD sensitivity (Pfaffenrot & Koopmans, 2022; Schulz et al., 2020). The venous signal was further reduced through the downstream exchange of saturated water from tissue to the veins, therefore resulting in increased sensitivity to the more spatially‐specific arterial and capillary CBV change (arterial blood contrast; ABC). The reduced sensitivity to the (negative) tissue CBV change and the additive effect of residual BOLD resulted in comparable sensitivity to standard BOLD methods (Schulz et al., 2020). This saturation‐based approach requires a short TE and results in increased SAR deposition, which make application at UHF and at high‐resolution challenging.

Here, we develop a magnetization‐preparation method that flexibly combines segmented BOLD‐weighted 3D‐EPIs (Poser et al., 2010; van der Zwaag et al., 2012) with a partial attenuation of the macrovascular venous and tissue signal thus increasing sensitivity to arterial and capillary CBV contributions. We leverage this efficient saturation scheme to derive both MT‐weighted BOLD as well as a microvasculature‐dominated CBV signal at submillimeter resolution at 7 T in humans. We subsequently examine the sensitivity and specificity of these fMRI methods.

## 2 MATERIALS AND METHODS

To create a time‐efficient ABC‐weighted fMRI method in the SAR‐constrained 7 T environment, we first combined and tested a sparsely‐repeated on‐resonance MT train with a center‐slice‐out, submillimeter 3D‐EPI. Additionally, an interleaved ABC‐ and BOLD‐ experiment was carried out to isolate the ABC component.

### 2.1 ABC‐weighted sequence development

#### 2.1.1 Pulse‐train development

On‐resonance rectangular pulse‐trains with 0‐net flip‐angle can efficiently saturate a selected bandwidth of *T*_2_ values in relation to the *B*_1_ amplitude (Forster et al., 1995; Hu et al., 1992; van Gelderen et al., 2017). It was empirically found that at 95% SAR at 7 T, a pulse‐train consisting of seven phase‐modulated, nonslice‐selective, rectangular subpulses with *B*_1_ = 10 μT (total duration = 6 ms; Priovoulos, Oliveira, et al., 2021; Schulz et al., 2020) could be repeated every 387 ms, thus allowing time for the readout of a 3D‐EPI segment.

**FIGURE 1:**
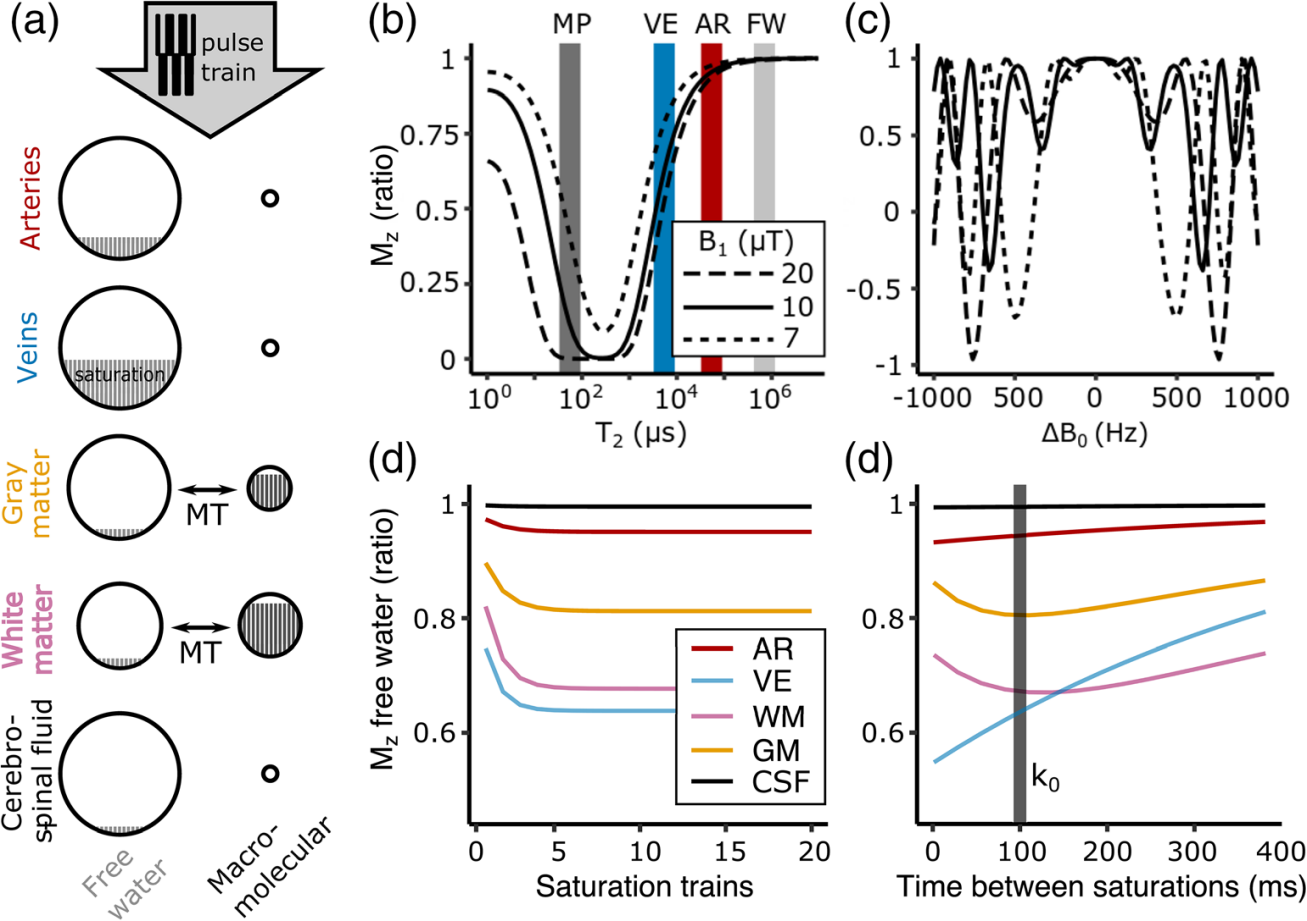
(a) Five signal compartments were considered. Arteries, veins, and cerebrospinal fluid were modeled as perfect liquids (i.e., minimal macromolecular pool) but with distinct *T*_2_ values. Gray and white matter were assumed to have a sizeable macromolecular pool which, following saturation (gray bands), exchanges magnetization with the free water pool. (b) Simulated effects of a single pulse‐train at different amplitudes (duration: 6 ms) on the longitudinal magnetization *M*_z_, assuming on‐resonance. AR, arterial blood (red); FW, free water pool (light gray); MP, macromolecular pool (dark gray); VE, venous blood (blue). With a single 10 μΤ pulse, the MP (*T*_2_ = 70 μs) is 92.5% saturated, and the VE compartment (*T*_2_ = 7 ms; Yacoub et al., 2001) loses 37% of its signal, while the AR (*T*_2_ = 67.5 ms) and FW (*T*_2_ = 900 ms) compartments experience minimal signal attenuation. (c) Effect of a single pulse‐train on *M*_z_ across offset frequencies for free water. Close to resonance no saturation occurs. (d) Effect of successive pulse‐trains on the free water signal‐reduction buildup. After <5 trains signal reduction is maximized. The signal is plotted for the time we intended to sample the center of *k*‐space (*k*_0_ = 100 ms). (e) Simulated free‐water *M*_z_ decay from *t* = 0 after the pulse‐train until its repetition (*t* = 381 ms). A biexponential behavior is observed in WM and GM due to the coupled macromolecular pool. The center of *k*‐space was sampled at the point of maximum AR/GM saturation difference (gray band at 100 ms; GM = 20% signal suppression, WM saturation = 33%, VE = 42%, AR = 6.5%, and FW = minimal).

To examine the direct and indirect (MT) signal reduction resulting from this pulse‐train, we simulated its effect assuming our signal consisted of compartments with distinct *T*_2_ and *T*_1_ values, namely venous (*T*_2_ = 7 ms, *T*_1_ = 2500 ms), arterial (*T*_2_ = 67.5 ms, *T*_1_ = 2500 ms), cerebrospinal fluid (*T*_2_ = 900 ms, *T*_1_ = 2500 ms), and extravascular tissue. The tissue (GM and WM) was assumed to consist of a macromolecular (*T*_2_ = 70 μs, *T*_1_ = 500 ms) and a free water pool (*T*_2_ = 900 ms, *T*_1_ = 2500 ms; Figure 1a), with WM being characterized by a larger macromolecular to free water pool ratio compared to GM (30% and 15%; values taken from literature; Krishnamurthy et al., 2014; Markuerkiaga et al., 2016; Pfaffenrot et al., 2021; Spijkerman et al., 2018; van Gelderen et al., 2016; Yacoub et al., 2001). The longitudinal decay of the GM and WM was assumed to show both *T*_1_ relaxation effects and exchange effects (MT) between the macromolecular and free‐water pools.

#### 2.1.2 Direct saturation simulation

The direct saturation following a single pulse‐train was simulated using the Bloch equations by modeling the RF as a finely‐sampled (*d* = 5 μs) series of delta pulses for three *B*_1_ values (*B*_1_ = 7, 10, and 20 μT). At *B*_1_ = 10 μT (i.e., what was found to be empirically feasible), we estimated that a single train selectively attenuated the macromolecular pool by 92.5% and the venous compartment by 37% (Figure 1b), while close to resonance (<300 Hz), the effect on free water/cerebrospinal fluid or arterial blood was found to be minimal (Figure 1b,c and Figures S1 and S2). A higher amplitude pulse‐train minimally increases the venous and macromolecular pool attenuation at a cost of a roughly quadratic increase in SAR and a higher sensitivity to off‐resonance effects (Figure 1b).

#### 2.1.3 Magnetization‐transfer simulation

With the repetition of the pulse‐train, the system experiences a saturation buildup. To model this, the free‐recovery solution for the Bloch equations was expanded to include a coupling term in the longitudinal domain to capture both the *T*_1_ relaxation and between‐pool exchange (Gochberg et al., 1997; van Gelderen et al., 2016; Zimmerman & Brittin, 1957), thus behaving in a biexponential manner:

$$S_{z}^{f}\left(t\right)=\frac{{1-M}_{z}^{f}\left(t\right)}{M_{z}^{f}\left(\infty \right)}=a_{1}*e^{-\lambda_{1}*t}+a_{2}*e^{-\lambda_{2}*t}\;$$

with

$$2*\lambda_{1,2}=R_{1m}+R_{1f}+k_{m}+k_{f}\pm \sqrt{{\left(R_{1m}-R_{1f}+k_{m}-k_{f}\right)}^{2}-4*k_{m}*k_{f}},$$

$$a_{1,2}=\pm \frac{S_{z}^{f}\left(0\right)*\left(R_{1f}+k_{f}-\lambda_{2,1}\right)-S_{z}^{m}\left(0\right)*k_{m}}{\lambda_{1}-\lambda_{2}}$$

and

$$\left(1-f\right)*k_{f}=f*k_{m}$$

where $M_{z}^{f}\;$ is the longitudinal magnetization of the free water pool (i.e., what is measured), $S_{z}^{f}$ is the fractional saturation of the free pool, $R_{1f},R_{1m}$ are the relaxation rates of the free and macromolecular pools in the absence of exchange, $k_{f},k_{m}$ are the exchange rates from the free and macromolecular pool, and f is the macromolecular‐to‐free water pool ratio. $\lambda_{1,2}$ reflect relaxation and exchange rates of the tissue, with the amplitudes $a_{1,2}$ reflecting the $M_{z}\;$ given the earlier pulse‐trains and longitudinal decay in between.

Modeling the longitudinal magnetization following each pulse‐train showed that the saturation buildup asymptotically approaches a maximum after approximately five pulse‐trains (Figure 1d). In the period between successive pulse‐trains, a longitudinal steady‐state is not reached. Instead, the simulated longitudinal magnetization shows a biexponential behavior in tissue with a coupled macromolecular pool (Figure 1e). This suggests that maximum MT contrast can be achieved with judicious traversing of the *k*‐space so that the center of the *k*‐space is sampled close to the point of maximum GM/arterial contrast. The maximum GM/arterial saturation difference was estimated to be at 100 ms following the pulse‐train (GM = 20% signal suppression). At this time point, the WM signal reduction was estimated at 33%, the venous at 42%, the arterial at 6.5%, and the cerebrospinal fluid minimal. The simulation code can be found at https://github.com/npriov/saturation_preparation_sim/blob/main/ABCfmri_sims.R.

#### 2.1.4 ABC‐weighted sequence implementation

**FIGURE 2:**
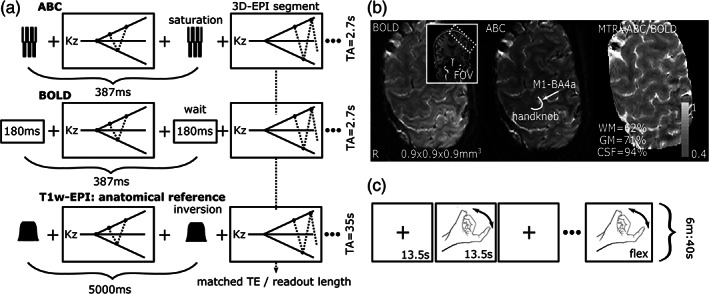
(a) Overview of ABC, BOLD, and *T*_1_w anatomical‐reference using the same segmented 3D‐EPI. (b) BOLD, ABC, and MTR = ABC/BOLD at the handknob (primary motor cortex). (c) Two counterbalanced runs of a finger‐flexing task were recorded, where participants were instructed to flex their right index finger without touching their palm or to rest while fixating on a cross.

A high‐resolution, *k*_z_‐segmented 3D‐EPI (FOV = 120 × 131 × 31 mm^3^, voxel‐size = 0.9 mm isotropic, TA/TR/TE = 2700/52/18 ms, flip‐angle = 20°, SENSE_y/z_ = 3.5/1.5, partial‐Fourier_y/z_ = 0.7, readout segment‐duration = 218 ms) interleaved with a pulsed saturation train (*T*_saturation‐to‐saturation_ = 387 ms), was set up on a 7 T scanner (Philips Achieva with an 8Tx/32Rx whole‐head coil [Nova Medical]; transmit channels set in quadrature mode). To maximize MT‐weighting, the 3D‐volume was sampled in a center‐slice out manner in the *k*_z_ direction (Figure 2a). A delay between the saturation pulse and the readout was added so that the *k*_0_ point of the *k*‐space was sampled close to the point of maximum saturation difference for GM/arteries (100 ms).

To examine the effect of the saturation in‐vivo in WM, GM, cerebrospinal fluid, veins and arteries, we acquired 3D‐EPI data from one participant in a pilot session with ABC‐ and BOLD‐weighting. Conventional BOLD‐weighting was achieved by replacing the pulsed saturation with an equivalent waiting time (Figure 2a). An MT ratio image was generated (MTR = ABC/BOLD; Figure 2b). Only the images after the initial 40 shots were included in the MT ratio map to ensure that the maximum saturation buildup had been reached. On that map, WM, GM, and cerebrospinal fluid were visually separable and we used it to manually define areas of interest (ROIs; NP). On the mean BOLD image, the signal hypointensities within the brain (that represent venous voxels) were segmented using a signal intensity ‐ gradient magnitude plot (Segmentator; Gulban et al., 2018). Additionally, an MP2RAGE scan was acquired (FOV = 230 × 230 × 186 mm^3^, TE/TR = 2.3/6.2 ms, TI_1_/TI_2_/TR_shot_ = 800/2700/5500 ms, flip‐angle = 7/5°, voxel‐size = 0.8 × 0.8 × 0.8 mm^3^, SENSE_y/z_ = 1.8/1.8, TA = 10 min) to facilitate arterial definition (Caan et al., 2019). A maximum intensity projection transform of the second inversion image from the MP2RAGE (INV2) was used to define an arterial ROI (LAYNII2.1; Huber, Poser, Bandettini, et al., 2021). The mean saturation values for the GM, WM, cerebrospinal fluid, venous, and arterial ROIs were extracted.

### 2.2 Data acquisition

Six participants (22–34 years old) performed two counterbalanced runs of a finger‐flexing task (right index finger, ON = 13.5 s, OFF = 13.5 s, run‐duration = 6 min:40 s) (Huber et al., 2017), while ABC‐ or BOLD‐weighted (same protocol as above; ABC‐ or BOLD‐weighting achieved with a saturation‐pulse or an equilength wait) images were recorded (Figure 2a,c). A matched *T*_1_‐weighted 3D‐EPI (Kashyap et al., 2018; van der Zwaag et al., 2018) was obtained by replacing the saturation train with an adiabatic inversion pulse (*TI* = 1100 ms) to use as a distortion‐matched anatomical reference (Figure 2a). The 3D‐EPIs were placed approximately perpendicular to the left primary motor cortex to optimally visualize the handknob (Figure 2b). The *B*_1_ amplitude was monitored during the scan session through the acquisition of a DREAM *B*_1_+ map (FOV = 224 × 224 × 168 mm^3^, voxel‐size = 3.5 mm^3^, TR/TE = 6/3 ms, flip angle = 7°) to ensure good fidelity to the nominal amplitude of the saturation pulses. The long‐term SAR limit was successfully observed in all six participants (SAR < 95%). For two participants, the session was repeated on a different day to examine the reproducibility of the results. For one participant, noise scans were acquired for both ABC and BOLD‐weighted 3D‐EPIs by turning off the RF and gradients. These noise scans were used to estimate the image SNR (SNR = Mean_3D‐EPI Amplitude_/Std_Noise scan_). Similarly, 50 3D‐EPI volumes were acquired during fixation for both ABC and BOLD‐weighted 3D‐EPIs, through which the tSNR was calculated (temporal SNR = Timeseries Mean_3D‐EPI Amplitude_/Timeseries Std_3D‐EPI Amplitude_). All participants provided written informed consent as approved by the ethics committee of the Amsterdam University Medical Centre.

### 2.3 Data analysis

The fMRI data were motion‐corrected and registered to the distortion‐matched *T*_1_‐weighted‐EPI with a six degrees‐of‐freedom transform. A single interpolation combining the motion‐correction and the *T*_1_‐weighted‐EPI transforms was employed to register the fMRI data to anatomical space (ANTs 2.1; Avants et al., 2011). A GLM (finger‐tapping > rest) was fitted (FSL6.0.1) and the percent signal change was calculated. A manual ROI was drawn on the anterior bank of the central sulcus, encompassing BA4a. This ROI is referred to here as M1. Eleven cortical depth profiles of the GM from WM to pial surface were extracted from the M1 ROI (LAYNII2.1; Huber, Poser, Bandettini, et al., 2021). Additionally, we segmented the GM throughout the whole slab (Segmentator; Gulban et al., 2018) and binned the active voxels into three cortical depths to examine the z‐stat distribution across brain. Finally, we examined the venous activation reduction within the venous ROI (drawn as described above), masked with the significantly active voxels during the BOLD acquisition.

### 2.4 Isolated‐ABC sequence implementation

**FIGURE 3:**
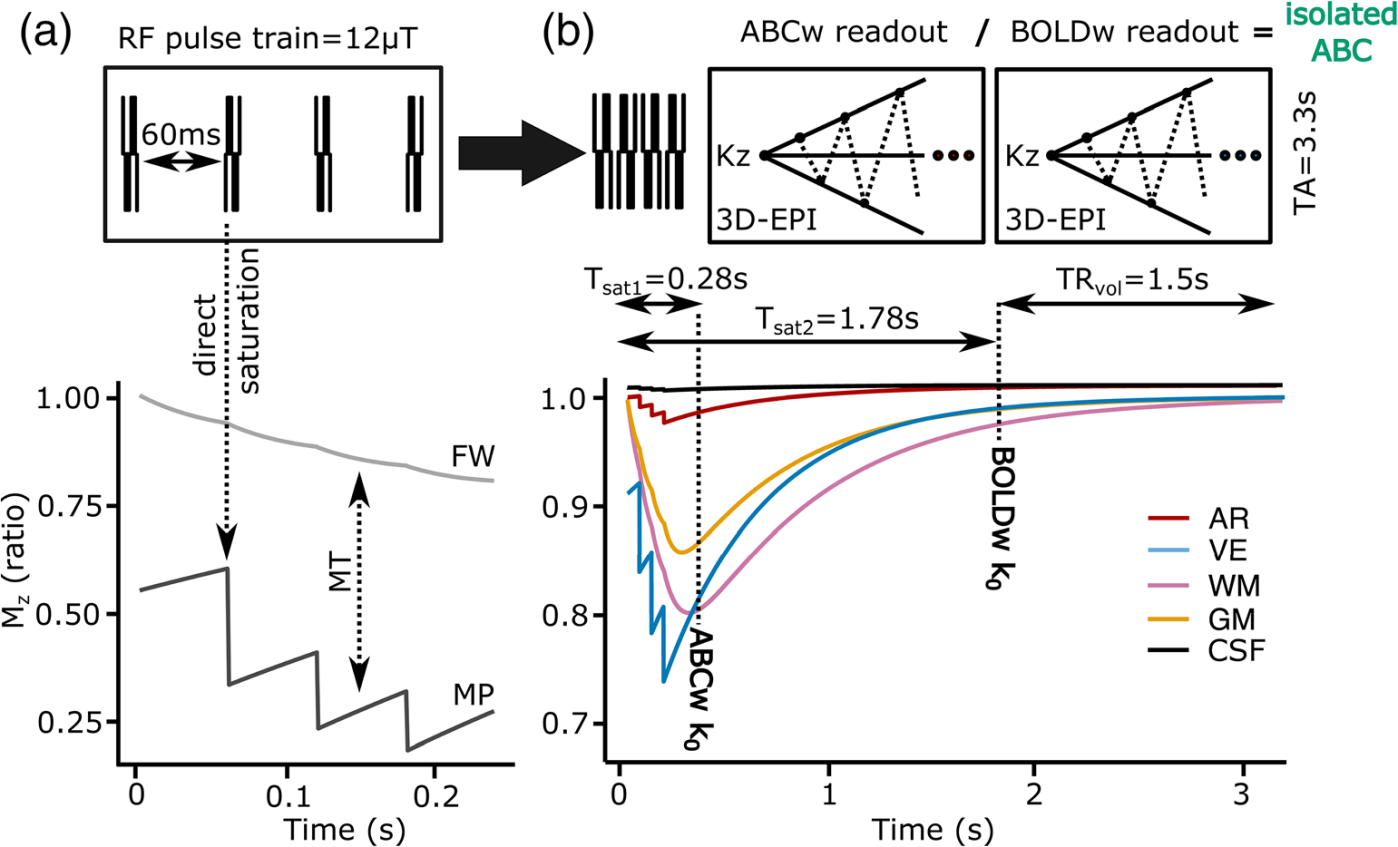
(a) A 12 μT pulse‐train interleaved with cross‐relaxation periods enhances tissue‐specific saturation from a single pulse‐train repetition. (b) The center‐slice‐out ordering scheme brought close to the first *k*_0_ at the maximum gray‐matter saturation point (ABC) and the second *k*_0_ close to the longitudinal equilibrium (BOLD), as shown through coupled‐compartment simulations.

An additional sequence was set up to isolate the ABC contrast from BOLD contamination. A rectangular, on‐resonance phase‐modulated pulse‐train (16 subpulses, *B*_1_ = 12 μT) was followed by two pairwise‐interleaved 3D‐EPI readouts (FOV = 140 × 141 × 20 mm^3^, voxel‐size = 0.8 × 0.8 × 1.5 mm^3^, TE/TR/TA_volume_/TA_total_ = 19/67/1500/3300 ms, flip‐angle = 20°, SENSE_y/z_ = 2.7/1, partial‐Fourier_y_ = 0.7) conceptually similar to VASO implementations (Huber et al., 2014). To maximize MT‐weighting, the pulse‐train was divided into four trains with 60 ms of cross‐relaxation in between. This lengthy but sparsely‐repeated magnetization preparation (*T*_saturation‐to‐saturation_ = 3.3 s) resulted in a saturation difference within the saturation‐to‐saturation period (coupled Bloch equations simulated in Figure 3b). We maximally profited from this saturation difference by acquiring the 3D‐EPIs in a center‐slice‐out fashion (Figure 3), so that the ABC *k*‐space_0_ point occurred close to the maximum GM/arterial saturation difference (ABC image), while the second readout was acquired close to the longitudinal equilibrium (BOLD image). This allowed isolating the ABC signal through BOLD normalization (isolated‐ABC = time‐matched ABC / BOLD).

### 2.5 Data acquisition and analysis

Five additional participants (23–31 years old) performed a hand‐flexing task (ON = 30 s, OFF = 30, run‐duration = 10 min), while the isolated‐ABC sequence was recorded. The FOV was placed in the left primary motor cortex.

Similar to the main experiment, the time‐matched ABC and BOLD fMRI data were motion‐corrected. The isolated‐ABC time series were extracted through ABC/BOLD division. A GLM (finger‐tapping > rest) was fitted for the time‐matched ABC, BOLD, and isolated‐ABC time series, the percent signal change was calculated and GM cortical depth profiles were extracted from a manually‐drawn M1 ROI. The z‐stat distribution within the M1 was extracted to examine the sensitivity of the isolated‐ABC compared to the time‐matched ABC and BOLD.

## 3 RESULTS

### 3.1 ABC‐weighted experiment

#### 3.1.1 Compartment‐specific signal reduction

**FIGURE 4:**
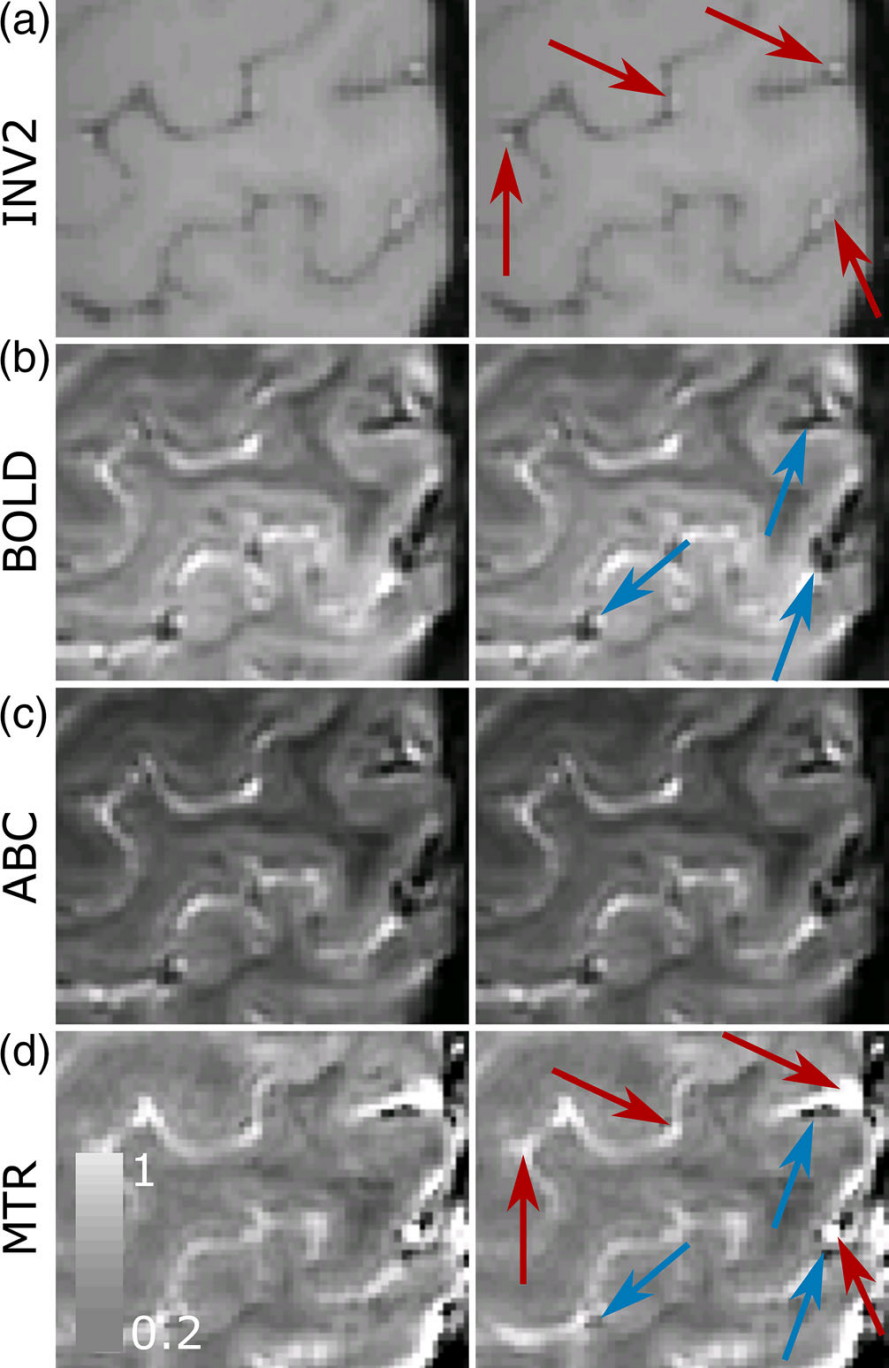
Axial slices at the level of M1 from one participant with venous and arterial visualization. (a) MP2RAGE INV2 image. (b) BOLD. (c) ABC. (d) Magnetization transfer ratio (MTR). The arteries can be seen as hyperintensities in INV2 (red arrows) and the veins as hypointensities in BOLD (blue arrows). In MTR, the veins, but not the arteries, are preferentially suppressed, confirming that the *T*_2_‐specific saturation train minimally affects the arterial signal.

The MT ratio map after maximum saturation buildup had been reached showed 38% signal suppression in WM, 29% in GM, and 6% in the cerebrospinal fluid (Figure 2b). These results were closely in line with our simulations (Figure 1d,e), though slightly higher, confirming experimentally that our combination of sparsely‐applied MT with center‐out readouts resulted in high signal reduction efficacy with limited direct saturation of longer‐*T*_2_ compartments. In the MT ratio image, the veins were preferentially suppressed by 51%, as shown in Figure 4. The arteries instead were largely unaffected (11% signal suppression), confirming the expected specificity of our saturation (Figure 4d). Overall, the addition of the pulse‐train attenuated the unwanted signal components for this EPI readout, but minimally affected the arterial signal. This signal suppression reduced the SNR across the brain (mean SNR_ABC_ [sd] = 108.74 [48.44] compared to mean SNR_BOLD_ [sd] = 150.74 [56.39]) and, as a result, the temporal‐SNR (mean temporal SNR_ABC_ [sd] = 12.52 [3.62] compared to Mean temporal‐SNR_BOLD_ [sd] = 17.17 [6.97]; Figure S3).

#### 3.1.2 BOLD‐response modulation

**FIGURE 5:**
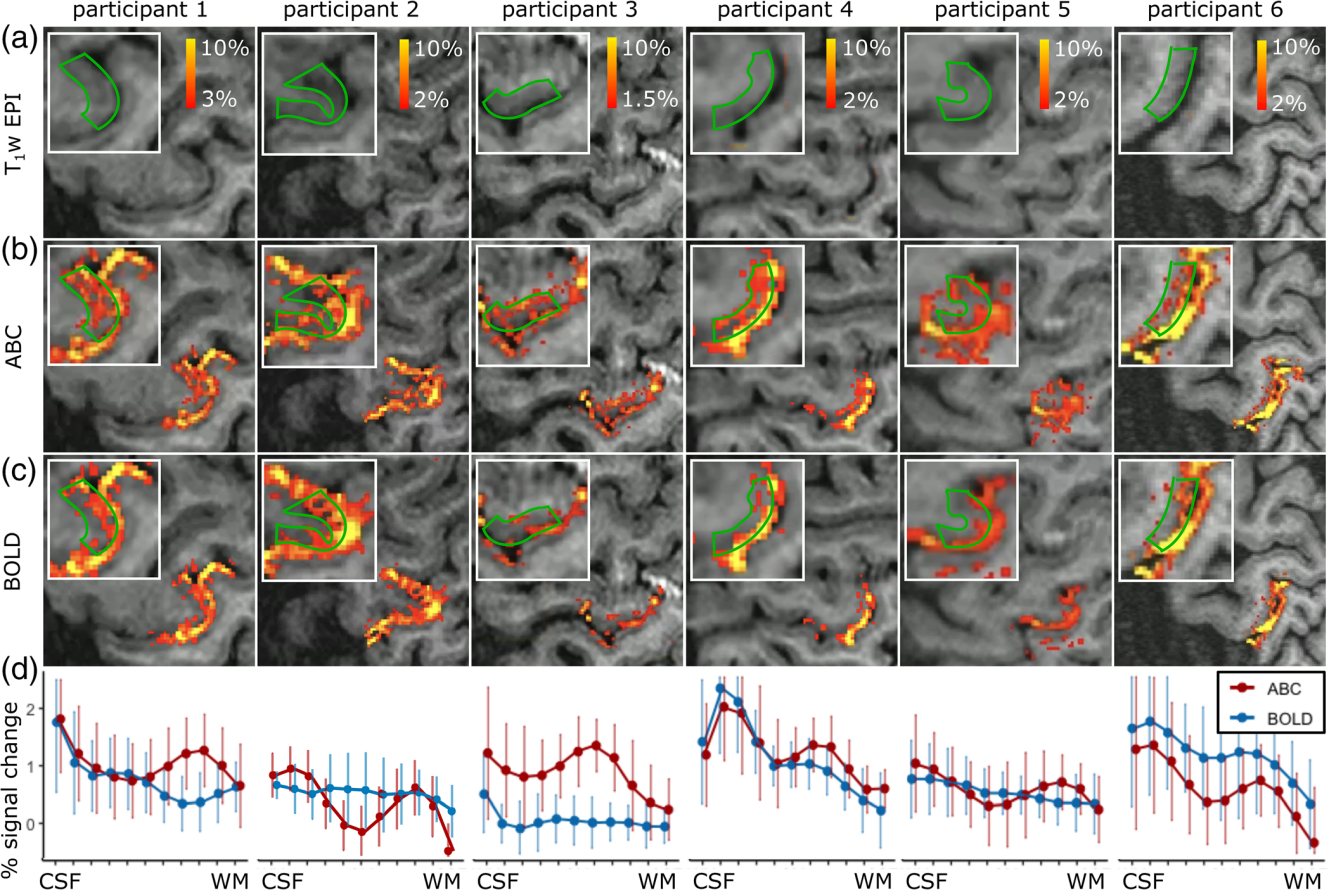
fMRI signal change in the left handknob during right index finger flexing (unsmoothed and masked for M1). Each column represents a participant. (a) Anatomical reference, (b) ABC, and (c) BOLD signal change. The white box highlights the M1. The green lines outline the cortex. (d) Mean cortical depth sampling within the M1 (red = ABC, blue = BOLD). The cortical depth profiles show increased signal change close to WM for ABC.

**FIGURE 6:**
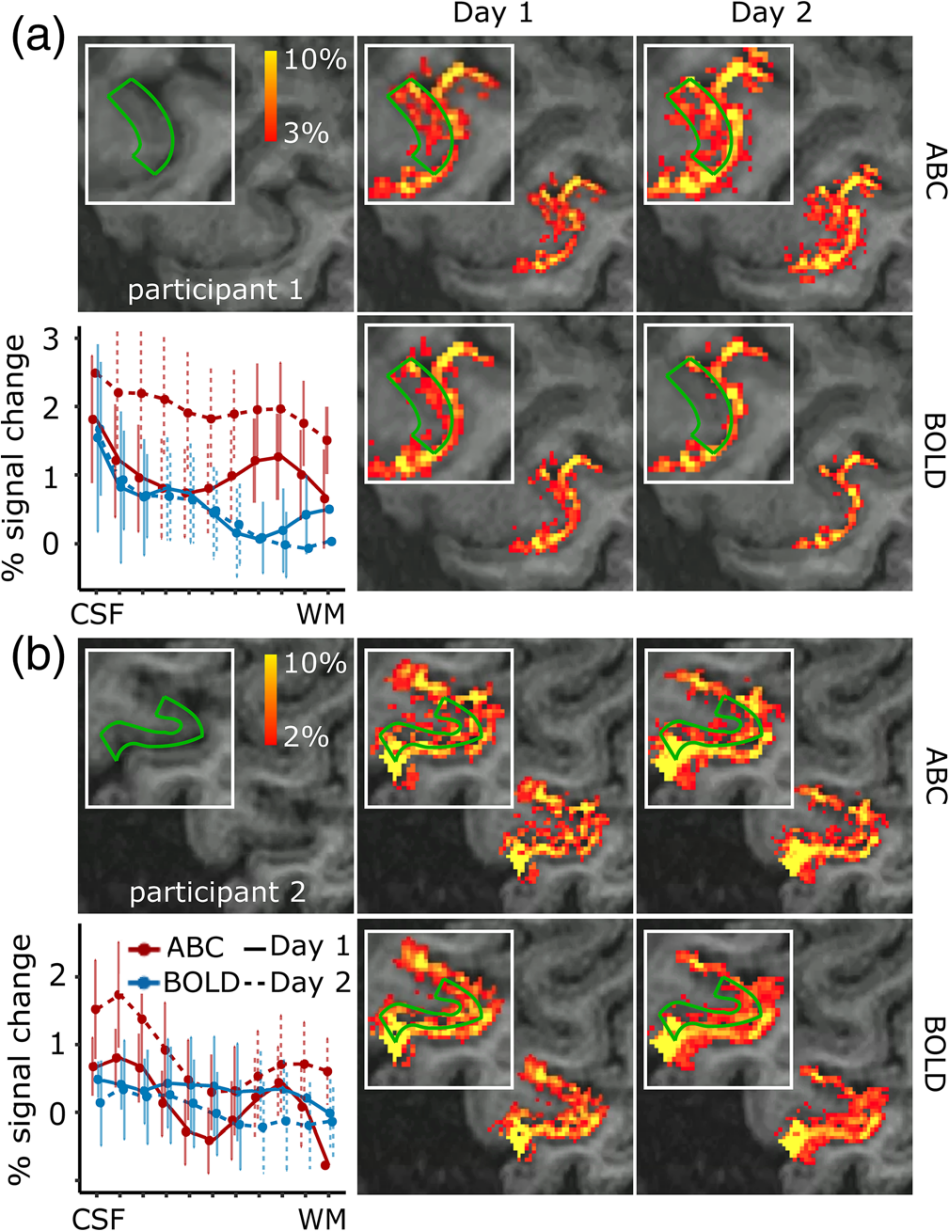
Reproducibility of the fMRI percent signal change in the left handknob during right index finger flexing. (a, b) Participants 1 and 2. M1 slices with unsmoothed percent signal change overlaid and cortical depth profiles for ABC and BOLD. The white box highlights the M1. Note that the participants showed a similar response pattern in both days, with the deep GM activation stripe in ABC being particularly obvious in participant 1.

**FIGURE 7:**
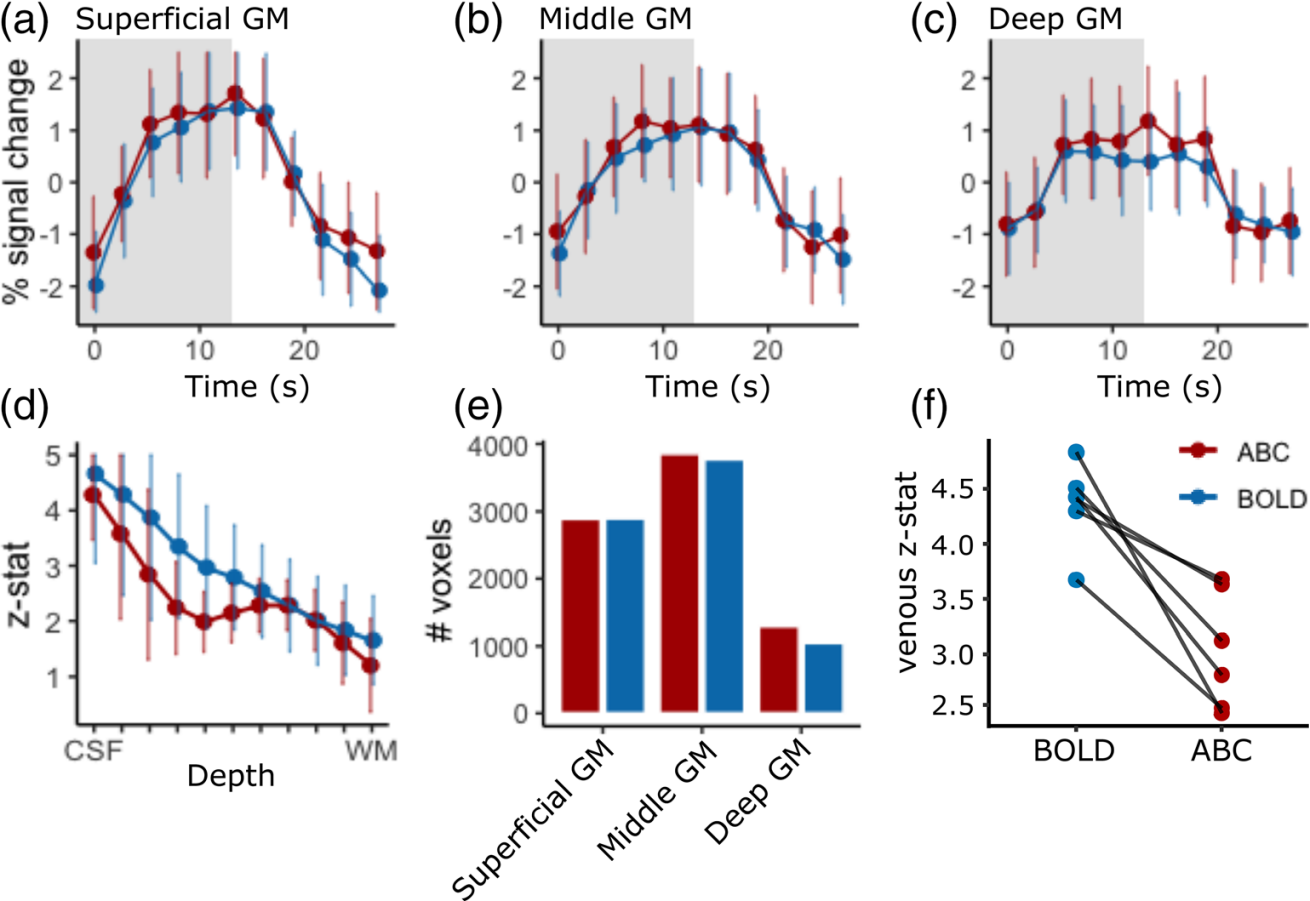
(a–c) Mean timecourse (gray‐band = stimulus) within‐group for superficial (a), middle (b) and deep GM (c) (red = ABC, blue = BOLD). (d) Mean z‐stat cortical depth values within the M1 within‐group. (e) Mean number of active voxels within‐group (only voxels *p* < .05 included) for superficial, middle, and deep GM. ABC shows similar sensitivity to BOLD. (f) Mean z‐stat values within the venous ROI. ABC shows reduced venous sensitivity.

Cortical depth sampling of the M1 region during finger flexing showed an increased percent signal change close to WM for ABC compared to BOLD, resulting in an activation bump in deep GM (peak percent signal change ranging from 0.75% to 1.3% between participants in deep GM) that was visually obvious in the ABC data (Figure 5a–d; unsmoothed percent signal change masked for M1; for percent signal change and z‐stat maps across multiple slices see Figure S4). Note that, as expected, both the ABC and BOLD cortical depth profiles show the pial bias associated with BOLD‐sensitivity. Examining the z‐stat cortical profile, as an additional sensitivity measure, showed similar results across individuals (Figure S5). Importantly, the ABC response was reproducible on different days, with a consistent activation pattern (Figure 6). The average ABC timecourse was similar to the BOLD (Figure 7a–c). Overall, these results suggest that our magnetization preparation modulates the BOLD response.

#### 3.1.3 ABC sensitivity

The mean cortical depth response of ABC across participants within M1 showed similar sensitivity to BOLD with a small *z*‐score reduction in mid‐GM (Figure 7d). The reduction in *z*‐score likely relates to the lower absolute signal changes in ABC. The mean *z*‐score cortical depth response profile was consistent throughout a leave‐one‐out analysis, suggesting this profile is not dependent to a single outlier (Figure S6). Examining the z‐stat distribution across the whole slab as an overall measure of sensitivity, showed that the mean number of active voxels (*p* < .05) was similar between BOLD and ABC, implying similar sensitivity, with a slight increase for ABC active voxels in deep GM (Figure 7e). Within the venous ROI, ABC consistently showed reduced z‐stat values (median_z‐stat_ = 2.97, IQR = [2.59–3.51]) compared to BOLD (median_z‐stat_ = 4.41, IQR = [4.32–4.49]; Figure 7f), confirming that the magnetization preparation reduced venous sensitivity.

### 3.2 Isolated‐ABC experiment

#### 3.2.1 Sparse‐saturation performance

Despite the sparse saturation used in the isolated‐ABC setup, MTR images with good image quality and GM/WM contrast could be extracted from the time‐matched ABC/BOLD division, at a submillimeter in‐plane resolution and within 3.3 s (Figure S7). The suppression factor was decreased compared to the more rapidly‐repeated saturation used in the initial experiment, in line with our simulations (MTR_GM_ = 82%, MTR_WM_ = 75%). The pulse‐train reduced tSNR (mean_time‐matched ABC_ [sd] = 21.8 [8.4]) compared to time‐matched BOLD: (mean_time‐matched BOLD_ [sd] = 25.7 [9.9]). The isolated‐ABC showed further reduced tSNR but comparable to the time‐matched ABC (mean_isolated‐ABC_ [sd] = 18.6 [7.1]; Figure S7).

#### 3.2.2 Cerebral‐blood‐volume response using isolated‐ABC

**FIGURE 8:**
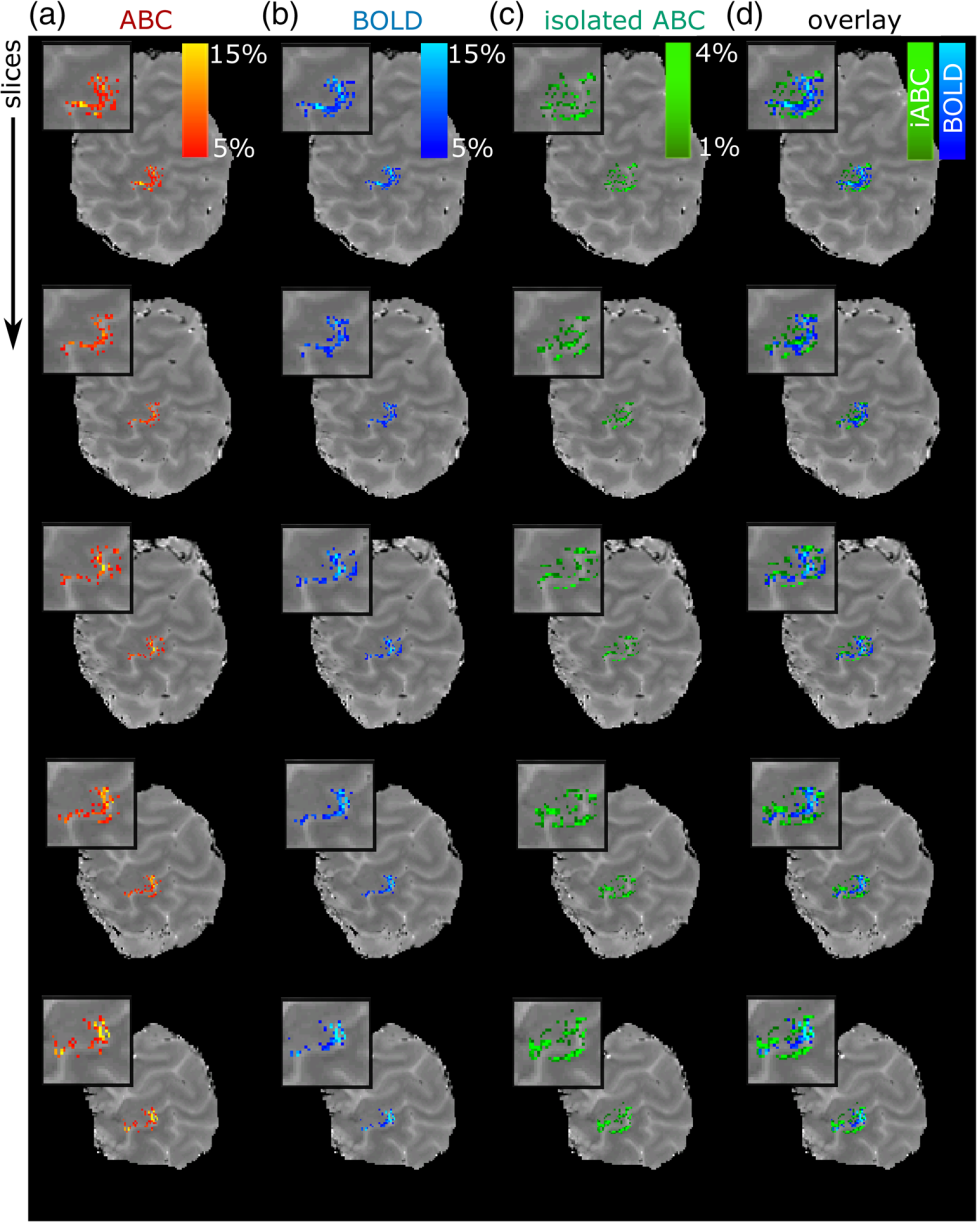
fMRI signal change in the left handknob during right index finger flexing (unsmoothed and masked for M1/S1) in one individual along successive slices (rows). (a) ABC. (b) BOLD (thresholded at 5%). (c) Isolated ABC signal change (thresholded at 1%). (d) Overlay between isolated ABC (green) and BOLD (blue). The box highlights the M1/S1. Note the M1/S1 separation in the functional response of isolated ABC compared to BOLD.

**FIGURE 9:**
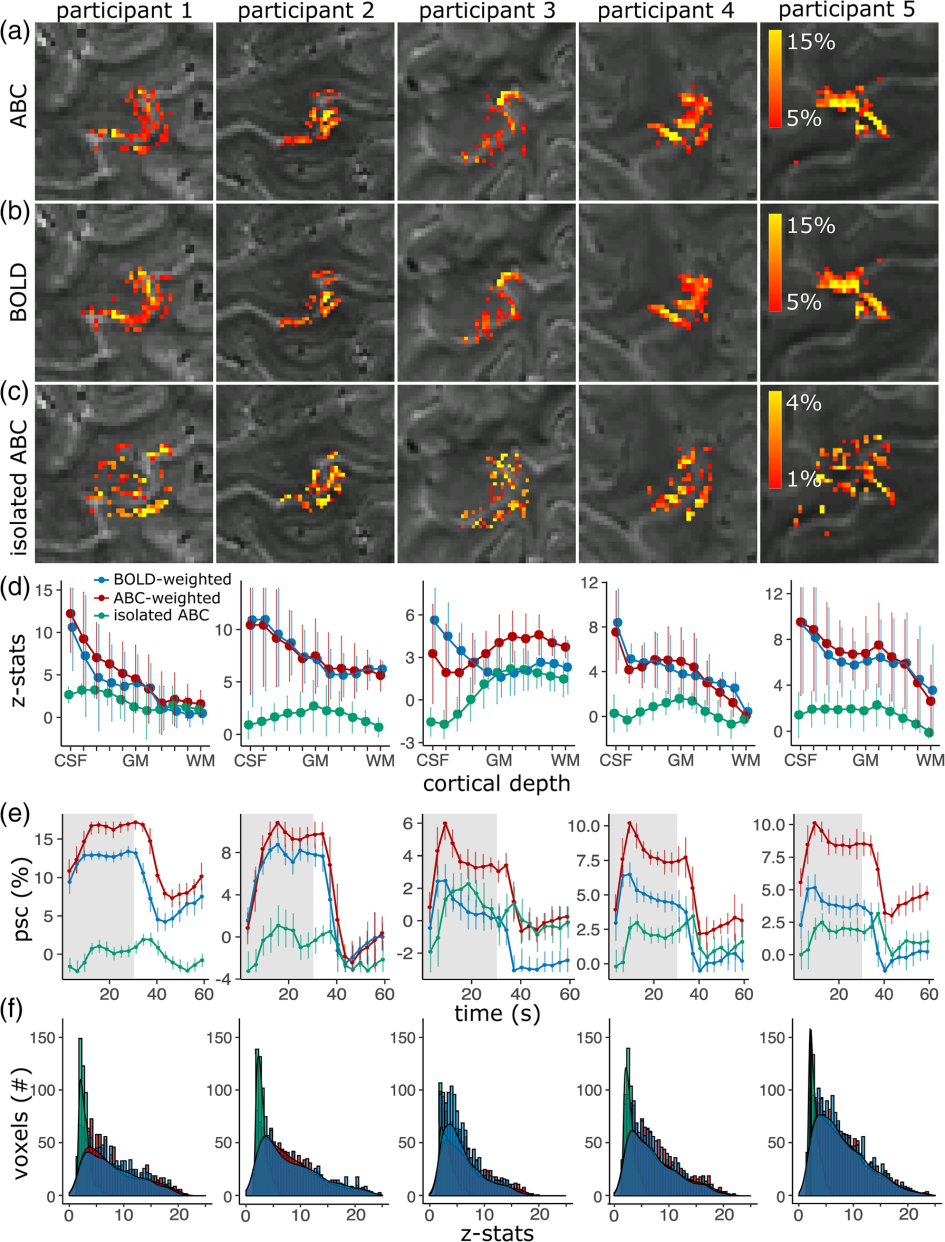
(a–c) fMRI signal change in the left handknob during right index hand flexing (unsmoothed and masked for M1/S1) for all participants (columns). (a) ABC. (b) BOLD (thresholded at 5%). (c) Isolated ABC signal change (thresholded at 1%). Distinct S1/M1 gray‐matter responses can be detected with isolated‐ABC compared to BOLD. (d) Cortical depth analysis. The isolated‐ABC shows reduced pial‐surface bias. (e) Mean timecourse (gray‐band = stimulus). (f) Comparison of z‐stat distribution of significant voxels (*p* < .05) in M1 (red = time‐matched ABC, blue = time‐matched BOLD, and green = isolated‐ABC).

The isolated‐ABC, a measure presumed to be mostly arterial CBV‐weighted, showed a consistent functional response to the task, largely constrained in the M1 and S1 GM, with limited activation in the surface vessels (visualized in Figure 8 across slices and Figure 9 across individuals). This was in contrast to the pial‐surface‐biased time‐matched BOLD response that did not separate the S1 and M1 areas on opposing banks of the central sulcus (Figure 8a–e). A cortical‐depth analysis confirmed a much‐reduced pial‐surface‐bias in isolated‐ABC (Figure 9f). For the used temporal resolution, the isolated‐ABC timecourse was similar to the time‐matched BOLD (Figure 9g).

The time‐matched ABC response, from which the isolated‐ABC signal was extracted, similarly showed a small signal increase in deeper cortical depths compared to the BOLD (Figure 9f). This modulation was qualitatively weaker compared to the initial ABC experiment, as expected from the decreased saturation factor, but sufficient to produce a consistent functional signal in the isolated‐ABC.

While the sensitivity of the time‐matched ABC and BOLD was similar (M1 z‐stat in significant voxels (*p* < .05) across‐group: mean_ABC_ [sd] = 7.51 [4.61]; mean_BOLD_ [sd] = 7.57 [4.70]; Figure 9f), the sensitivity of the isolated‐ABC component was lower (mean_isolated‐ABC_ [sd] = 2.94 [1.06]), though detectable in all individuals within a single 10 min functional run.

## 4 DISCUSSION

Improving the spatial specificity of fMRI is likely to have widespread neuroscientific applications in determining the cortical input and output and, hence, the directionality of information flow in the brain. This target is frequently hindered by the current tradeoff between sensitivity and specificity in high‐resolution fMRI applications. Here, we show that pulsed saturation can be employed to isolate the microvasculature‐dominated arterial CBV response at high resolution at 7 T. We also show that combining this simple manipulation with typical BOLD contrast can increase intracortical contrast while retaining much of the sensitivity of high‐resolution BOLD fMRI.

In our approach, the selective saturation of the tissue but not the arteries (Figure 4) increased our sensitivity to the CBV changes due to arterial and capillary dilation, in line with previous research (Kim et al., 2008; Vazquez et al., 2006). This tissue attenuation further reduced our sensitivity to the downstream‐to‐the‐activation extravascular static dephasing around large veins that is inherent in *T*_2_*‐dominated EPIs (Fujita, 2001; Pfaffenrot & Koopmans, 2022). Finally, the pial‐biased venous response was suppressed through direct saturation as well as potentially through the downstream‐exchange of suppressed water protons from tissue to veins.

The combination of these functional effects when a saturation and a typical *T*_2_*‐weighted readout were employed resulted in a z‐stat reduction in the venous voxels (Figure 7f) and a distinct cortical depth profile that consistently showed increased intracortical contrast within the human hand knob, suggesting increased specificity compared to standard BOLD. These results are in agreement with previous experiments in the cat cortex, where increased laminar specificity was found following the introduction of a saturation scheme (Kim & Kim, 2010), as well as recent experiments in humans using a FLASH acquisition (Pfaffenrot & Koopmans, 2022). Importantly, interleaving an ABC and BOLD acquisition to isolate the CBV functional effect of the pulsed saturation without motion and run‐to‐run variability confounds, produced a signal largely contained within the GM and with minimal pial‐surface bias (Figures 8 and 9). These results are in line with the hypothesized microvasculature dominance of the arterial functional response (Kim et al., 2008).

MT‐weighted acquisitions are not trivial at 7 T due to the severe SAR restrictions (Mougin et al., 2010; Priovoulos et al., 2019). Here we introduced a framework that leveraged the slower longitudinal relaxation at UHF (Rane & Gore, 2013) and judicious traversing of the *k*‐space through center‐slice out readouts to create sufficient contrast from sparsely‐repeated pulse‐trains. Through simulations and acquisitions, we showed that relatively long intervals between MT‐pulse‐trains can be employed, while still retaining substantial signal attenuation. This framework facilitates fitting high‐resolution readouts within the SAR constraints and with minimal dead time at 7 T, compared to the typical MT‐imaging that requires a longitudinal steady‐state through the rapid repetition of pulse‐trains (Henkelman et al., 1993). Sparser saturation instead results in a longitudinal magnetization evolution within the period between successive pulse‐trains which we leveraged to extract the ABC component without the BOLD contamination: we combined an extensive magnetization preparation, consisting of several interleaved periods of direct saturation and cross‐relaxation, followed by center‐slice‐out acquisitions close to the point of maximum signal reduction and the point of longitudinal equilibrium respectively. This is a setup conceptually similar to VASO acquisitions (Huber et al., 2017).

The isolated‐ABC CBV component can be thought of as the opposite of VASO (Lu et al., 2003, 2013) since it focuses on the intravascular CBV change. Similar to tissue CBV, it may be possible to extract a quantitative arterial CBV measure through appropriate modeling (Kim et al., 2008), though the sensitivity of our approach to the exchange between intra‐ and extravascular spaces remains to be examined. The isolated‐ABC does not require blood‐nulling and its contrast mechanism is not sensitive to inflow effects. This may allow CBV fMRI regardless of the area of interest or intended coverage and resolution, something not trivial with alternative approaches. Our isolated‐ABC implementation showed sufficient sensitivity to detect a consistent cortical response following a 10 min run at submillimeter in‐plane resolution and with a similar acquisition time to typical VASO implementations (Oliveira et al., 2022). Note that the contrast‐to‐noise in our isolated‐ABC acquisition strategy can be further increased through more extensive magnetization preparation, potentially increasing sensitivity.

Intriguingly, we also show that the simple combination of saturation with a *T*_2_*‐weighted readout modulates the BOLD signal with minimal acquisition cost beyond a SAR increase and a slight SNR reduction. This adds an extra degree of freedom in fMRI signal optimization: the TE and tissue/venous suppression can be jointly optimized to, for example, increase contrast‐to‐noise and specificity in shorter TEs (Pfaffenrot & Koopmans, 2022; Schulz et al., 2020), thus increasing SNR and acquisition efficiency (a natural fit with spiral imaging; Kurban et al., 2022) or reduce the venous bias. In our current implementation and without the additional readout to negate BOLD contrast and thus isolate ABC, there is a sizable pial‐surface bias that renders the examination of cortical depth profiles challenging, similar to typical BOLD.

Our current approach has several drawbacks: the venous suppression implemented here is potentially confounded by the venous *T*_2_* dependence on blood oxygenation and, therefore, may be reduced during functional activation (Pfaffenrot & Koopmans, 2022). In practice, we did not observe this: for our pulse‐train the venous activation was predominantly suppressed, rather than increased. Careful calibration of the *T*_2_‐bandwidth of the saturation though may better target specific compartments of the venous vasculature. Additionally, we did not attempt a comparison with more established layer‐resolving fMRI techniques, the specificity of which has been confirmed with contrast‐enhanced fMRI (Huber, Poser, Kaas, et al., 2021; Jin & Kim, 2008). In the future, this is needed to validate the spatial specificity of ABC. Furthermore, this is a high SAR method which can be a limiting factor. Finally, our current implementation employs hard rectangular pulses, trading off increased sensitivity to *B*_1_/*B*_0_ inhomogeneities (Figure S2) for saturation efficiency. This may be problematic in areas like the frontal lobe or cerebellum (Priovoulos, Roos, et al., 2021). Adiabatic, pTX‐enabled, or off‐resonance RF pulses may offer a better tradeoff for high‐field application across the brain.

There are several additional factors to be considered in transferring ABC imaging across field strengths, compared to BOLD where the signal‐to‐noise and the *T*_2_* dependence are the main considerations (Raimondo et al., 2021): while the exchange rate between the macromolecular and free water pool is minimally affected by B_0_ (van Gelderen et al., 2017), the *T*_1_ relaxation of both pools is slower at higher field strength, facilitating MT. The MT advantage at UHF is offset by the roughly quadratic increase in SAR (Bottomley & Edelstein, 1981; Collins & Wang, 2011). Finally, the relevant range of *T*_2_ values that needs to be saturated is *B*_0_ dependent and therefore the pulse‐train needs to be adapted to the field‐strength.

## 5 CONCLUSION

In sum, we show that a saturation‐prepared fMRI method with reduced tissue and venous but not arterial sensitivity differentiated the intracortical fMRI response compared to typical BOLD. Isolating this response showed that it is restricted to GM in line with the expected arterial CBV sensitivity. Importantly, the user‐friendliness of this method in terms of implementation, temporal efficiency, and sensitivity renders it attractive for high‐resolution laminar fMRI and may have widespread applications in cognitive and clinical in‐vivo neuroscience in the future.

## CONFLICT OF INTEREST STATEMENT

The authors declare no conflict of interest.

## Supporting information


**DATA S1.** Supporting Information

## Data Availability

The data that support the findings of this study are available on request from the corresponding author. The data are not publicly available due to privacy or ethical restrictions. The code for the simulations is available on Github (see Materials and Methods).
