# Supplementary material for: Combining arterial blood contrast with BOLD increases fMRI intracortical contrast
Source: Hum Brain Mapp. 2023 Feb 10;44(6):2509–22. doi: 10.1002/hbm.26227 (PMC10028680; doi:10.1002/hbm.26227)
Supplement: Supplementary file 1 — DATA S1. Supporting Information [file HBM-44-2509-s001.docx]

**Supplementary material**

**
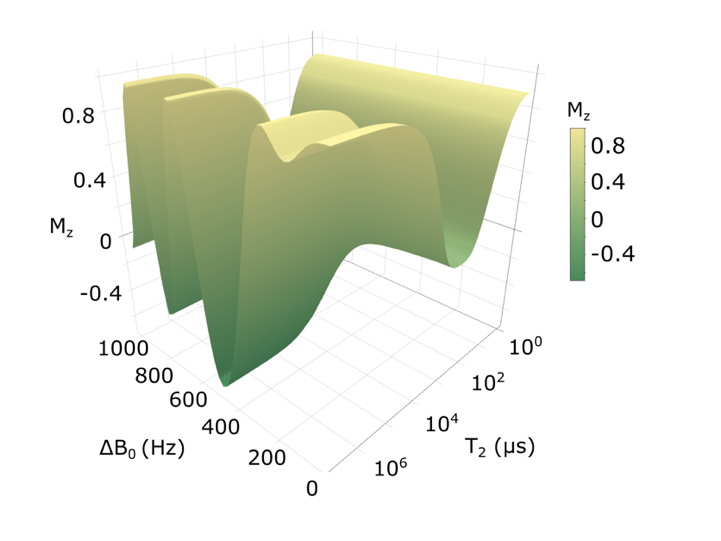
**

*Supplementary Figure 1: Simulated effects of a single phase-modulated saturation train (B_1_=10μT; duration: 6ms) on the longitudinal magnetization M_z_ as a function of T_2_ and frequency offset.*

*
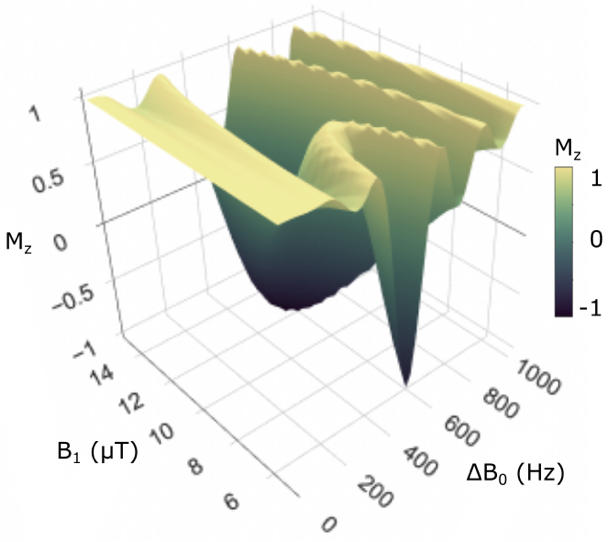
*

*Supplementary Figure 2: Simulated effects of a single phase-modulated saturation train (duration: 6ms) on the longitudinal magnetization M_z_ as a function of B_1_ and frequency offset. Note that within 200Hz from resonance, B_1_ variations affect the Mz only marginally.*

*
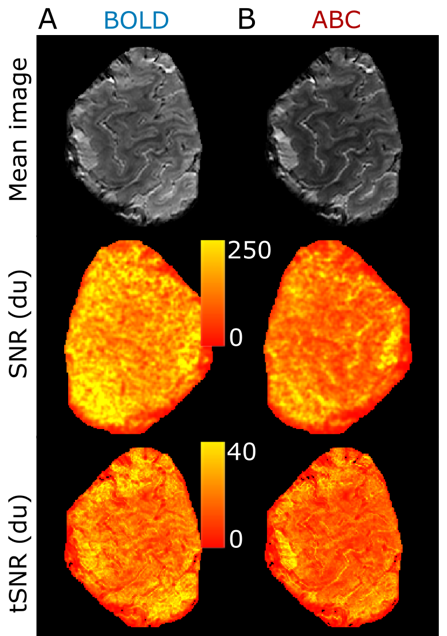
*

*Supplementary Figure 3: Axial slices of mean image (top), SNR (middle) and temporal-SNR (bottom) for BOLD (A) and ABC (B). Note that the suppression reduces SNR and subsequently the temporal SNR.*

*
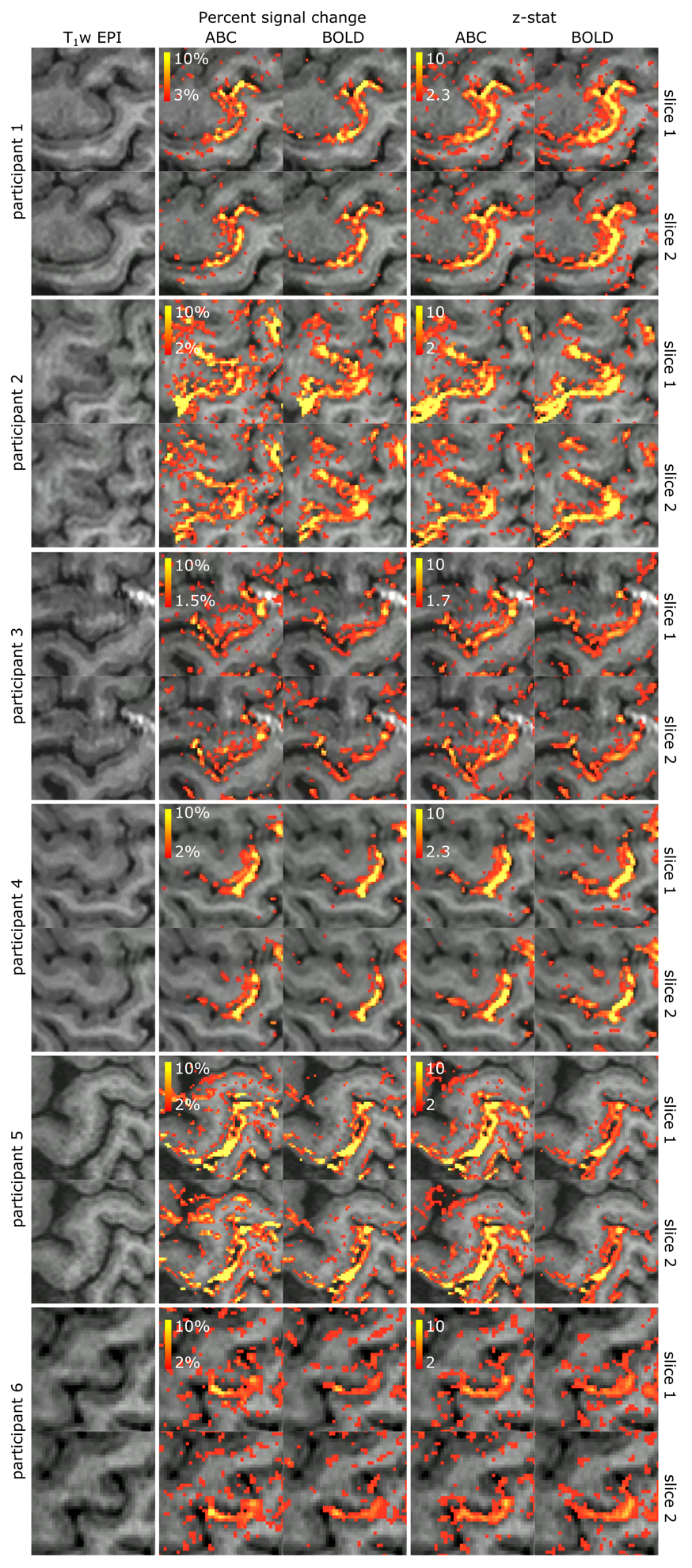
*

*Supplementary Figure 4: Individual fMRI activation in the left handknob during right index finger flexing; unsmoothed ABC and BOLD data at the level of M1 across two consecutive slices. Left column, anatomical reference. Middle columns, ABC and BOLD percent signal change. Right columns, ABC and BOLD z-stat maps. Every two rows represent a participant.*

**
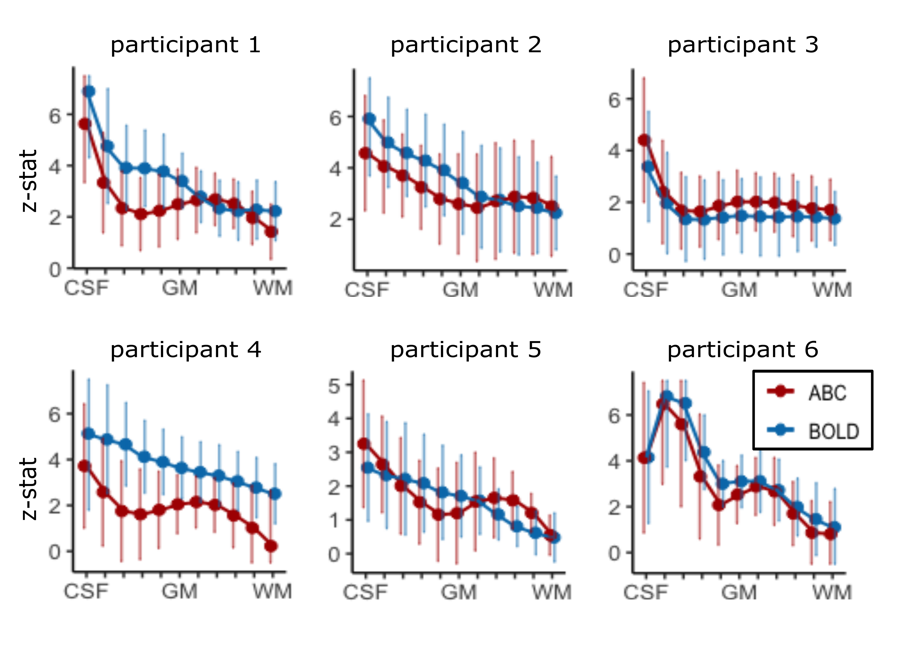
**

*Supplementary Figure 5: Individual z-stat cortical depth in the left handknob during right index finger flexing (all voxels within area-of-interest included). Each plot represents a participant. Red, ABC, Blue, BOLD. The cortical depth profiles show increased signal change close to WM for ABC.*


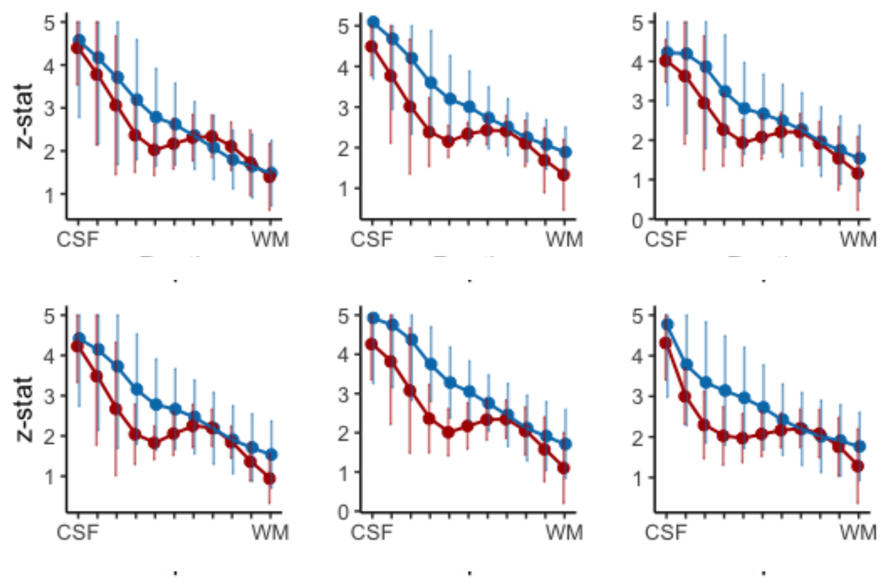


*Supplementary Figure 6: Mean z-stat cortical depth values within the M1-BA4a within group; leave one out analysis. Each plot represents the mean z-stat cortical depth response when one participant is left out. Red, ABC, Blue, BOLD.*


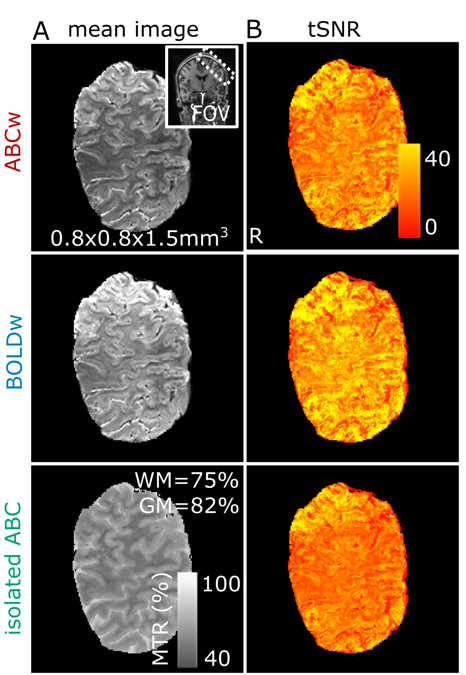


*Supplementary Figure 7: Isolated-ABC setup. A, Mean images (axial slice). B, Temporal-SNR (tSNR) images.*
